# Mice lacking the conserved transcription factor *Grainyhead-like 3* (*Grhl3*) display increased apposition of the frontal and parietal bones during embryonic development

**DOI:** 10.1186/s12861-016-0136-7

**Published:** 2016-10-18

**Authors:** Stephen J. Goldie, Benedicta D. Arhatari, Peter Anderson, Alana Auden, Darren D. Partridge, Stephen M. Jane, Sebastian Dworkin

**Affiliations:** 1 Department of Medicine, Monash University Central Clinical School, Prahran, VIC 3004 Australia; 2 ARC Centre of Excellence for Advanced Molecular Imaging, Department of Chemistry and Physics, La Trobe University, Melbourne, VIC 3086 Australia; 3 Australian Craniofacial Unit, Women and Children’s Hospital, Adelaide, SA 5005 Australia; 4 Faculty of Health Sciences, University of Adelaide, Adelaide, SA 5005 Australia; 5 Nanjing Medical University, Nanjing, People’s Republic of China; 6 Present address: Department of Physiology, Anatomy and Microbiology, La Trobe University, Melbourne, VIC 3086 Australia

**Keywords:** Frontal-parietal bone apposition, Craniosynostosis, Grainyhead-like, Grhl3, Transcription factors, Mouse models, Craniofacial

## Abstract

**Background:**

Increased apposition of the frontal and parietal bones of the skull during embryogenesis may be a risk factor for the subsequent development of premature skull fusion, or craniosynostosis. Human craniosynostosis is a prevalent, and often serious embryological and neonatal pathology. Other than known mutations in a small number of contributing genes, the aetiology of craniosynostosis is largely unknown. Therefore, the identification of novel genes which contribute to normal skull patterning, morphology and premature suture apposition is imperative, in order to fully understand the genetic regulation of cranial development.

**Results:**

Using advanced imaging techniques and quantitative measurement, we show that genetic deletion of the highly-conserved transcription factor *Grainyhead-like 3* (*Grhl3*) in mice (*Grhl3* ^*−/−*^) leads to decreased skull size, aberrant skull morphology and premature apposition of the coronal sutures during embryogenesis. Furthermore, *Grhl3* ^*−/−*^ mice also present with premature collagen deposition and osteoblast alignment at the sutures, and the physical interaction between the developing skull, and outermost covering of the brain (the dura mater), as well as the overlying dermis and subcutaneous tissue, appears compromised in embryos lacking *Grhl3*. Although *Grhl3* ^*−/−*^ mice die at birth, we investigated skull morphology and size in adult animals lacking one *Grhl3* allele (heterozygous; *Grhl3* ^*+/−*^), which are viable and fertile. We found that these adult mice also present with a smaller cranial cavity, suggestive of post-natal haploinsufficiency in the context of cranial development.

**Conclusions:**

Our findings show that our Grhl3 mice present with increased apposition of the frontal and parietal bones, suggesting that *Grhl3* may be involved in the developmental pathogenesis of craniosynostosis.

**Electronic supplementary material:**

The online version of this article (doi:10.1186/s12861-016-0136-7) contains supplementary material, which is available to authorized users.

## Background

Increased apposition of developing bones in the skull is an early event in the aetiology of craniosynostosis, the pathological condition whereby cranial sutures fuse prematurely. This may produce a spectrum of congenital deformities of the skull, and brain, and is estimated to affect 1:2,000–1:2,500 live births [1–3]. Depending on the pattern of affected sutures the resulting abnormalities may range from aesthetic anomalies to severe neurological deficiencies and impaired cranial growth.

Craniosynostosis can occur as an isolated anomaly or in conjunction with other congenital anomalies as part of a syndrome. Syndromic cases of the common occurring syndromes: Muencke, Crouzon, Apert and Pfeiffer have been found to result from mutations of the FGFR family of genes, where other anomalies of the skeleton co-exist. The aetiology of most non syndromic cases remains uncertain, but genetic mutations and epigenetic factors, including maternal drugs and diet are recognized [4].

Four major sutures exist in the mammalian skull, termed the sagittal, metopic, coronal, and lambdoid, and the correct temporal fusion of these is required to ensure that the embryonic and infant brain is able to grow, and correctly develop, within the cranial cavity. Many different combinations of anomalies affecting one or more sutures have been described in humans. Bilateral premature closure of the coronal sutures limits growth in the sagittal plane and results in brachycephaly (a skull shape shorter than typical for its’ species). Unilateral premature closure of a coronal suture results in asymmetrical distorted growth of the skull, termed plagiocephaly. The coronal suture is an embryologically unusual suture because it is the site of neural crest derived frontal bone and mesoderm derived parietal bone, and as such presents as an interesting embryological study of germ-layers and their derivatives. Defects in early development, particularly the establishment of neural crest-mesoderm interfaces at the suture boundary [5] as well as defects in the overlying surface ectoderm or underlying dura mater, may contribute to closer apposition of the parietal and frontal bones and premature coronal fusion.

Attempts to find specific genetic mutations resulting in craniosynostosis have yet to fully uncover the mechanisms by which premature suture fusion occurs in humans. Our studies have revealed a gene which may be a novel candidate for some of these conditions, the highly conserved transcription factor Grainyhead-like 3 (*Grhl3*), a member of the *Grainyhead-like* genes essential for normal development [6]. Deletion of *Grhl3* in mouse (*Grhl3* ^*−/−*^) results in neural tube defects [7], mimicking the human pathology spina bifida, as well as sporadic failed closure of the anterior neural tube, resulting in exencephaly in approximately 4 % of cases. This condition is exacerbated in embryos which lack both *Grhl3* and the related family member *Grhl2*, whereby embryos present with fully penetrant failure of anterior neural tube closure [8]. *Grhl3* ^*−/−*^ embryos also fail to develop a functioning epidermal barrier, and die immediately post-natally from trans-epidermal water loss and resulting dehydration [9]. Furthermore, *Grhl3* ^*−/−*^ embryos also present with numerous other surface ectoderm defects, namely defective wound healing and impaired cellular migration [10, 11], predisposition to squamous-cell carcinoma [12], as well as defective convergence-extension mediated migration [7], resulting in shorter, fatter embryos.

Here we present novel observations of the skull of *Grhl3* ^*−/−*^ embryos, as well as adult *Grhl3* ^*+/−*^mice, which reveal that loss of *Grhl3* results in impaired skull formation, and closer apposition of the parietal and frontal bones, with concomitant premature osteoblast alignment and organisation in embryos, and decreased cranial vault size in adults. Although important to note that we do not observe actual premature fusion of the frontal and parietal bones, due to embryonic lethality at E18.5, our observations indicate that loss of *Grhl3* is a novel genetic model of closer apposition of the parietal and frontal bones. These data suggest the possibility that abrogation of *Grhl3* function may underpin the aetiology of vertebrate craniosynostosis.

## Methods

### Animal breeding

All animal experiments were pre-approved by the Alfred-Monash University Research and Education Precinct (AMREP) Animal Ethics Committee (AEC), project #E1200/2012/M. For embryological analyses, *Grhl3* ^*+/−*^ mice were intercrossed, and detection of a vaginal plug was designated as embryonic day (E) 0.5. Embryos were collected at day 18.5 post fertilisation (E18.5), and euthanased by decapitation. In order to examine skulls from adult *Grhl3* ^*+/−*^ mice, adults were euthanased by CO_2_ administration. All animals were maintained on a C57/Bl6 background for a minimum of 24 generations, and female embryos/adults were used to both exclude the possibility of confounding hormonal influences on suture fusion/skull development, as well as to minimise the number of adult mice required.

### Micro X-ray computed tomography

Micro X-ray Computed Tomography (μXCT) measurements were conducted using an Xradia© micro XCT200 (Carl Zeiss X-ray Microscopy, Inc., USA). This uses a microfocus X-ray source with a rotating sample holder and an imaging detector system. The source consists of a closed X-ray tube with the tube voltage of 40 kV and a peak power of 10 W. One data acquisition set contained 361 equiangular projections over 180° providing a complete tomographic reconstruction. The exposure time was 8 s for each projection. The tomographic scan involved rotating the sample whilst recording transmission images on the charge-coupled device (CCD). Each projection image was corrected for the non-uniform illumination in the imaging system, determined by taking a reference image of the beam without sample. A filtered back-projection algorithm (TXM Reconstructor, Carl Zeiss X-ray Microscopy, Inc., USA) is used to obtain the 3D reconstructed image. The final three-dimensional reconstructed image size was 512 × 512 × 512 voxels. Two different set-ups were used in this study:

1. The first set-up was used to acquire the embryo samples, with an effective voxel size of 31 μm × 31 μm × 31 μm and a Field of View (FOV) of 16 mm × 16 mm × 16 mm.
2. The second set-up was to acquire adult female skull samples (*n* = 8 WT and *n* = 11 *Grhl3* ^*+/−*^) with an effective voxel size of 67 μm along each side and a FOV of 34 mm along each side.

We used the Avizo-6.2 software (Mercury Computer Systems Inc., France) for image segmentation of the embryo samples and TXM3DViewer (Carl Zeiss X-ray Microscopy, Inc. USA) for the 3D visualization and measurements. Measurements of the skull were made to find the maximum sagittal length of the skull from the tip of the nasal bones to the occiput, as well as the maximum sagittal length, height and width of the cranial cavity.

2D images of E18.5 embryos (*n* = 4 WT and *n* = 4 *Grhl3* ^*−/−*^) generated with the software to show the frontal and parietal bones from above were analysed to measure coronal suture area using ImageJ software [13]. Statistical analysis of measurements (Student’s *T*-Test) was performed using Prism GraphPad (San Diego, CA, USA).

### Morphometric quantitation of skull morphology

Skull landmarks used for quantitative analyses were as reported previously [14]. In brief, we utilised 7 of these landmarks for our measurements, these were: (1) Most anterior point of the nasal bone, medial region (mnsla), (2) Most superior point on the squamous temporal, intersection of the coronal suture, left side (lsqu), (3) Mid-point on the posterior margin of the foramen magnum, taken on squamous occipital (opi), (4) Most medial intersection of the frontal and parietal bones, taken on the parietal, left side (lfpi), (5) Most inferior portion of the cranial vault (cvi), (6) Most posterior point on the posterior extension of the forming squamosal, right side (rpsq), (7) Most posterior point on the posterior extension of the forming squamosal, left side (lpsq).

### Cartilage and bone histology

Heads from E18.5 mouse embryos were prepared for staining by fixation in 4 % Paraformaldehyde (PFA), decalcified in a solution of 10 % EDTA (w/v) for 24–48 h, and processed using standard histological procedures. Samples were then embedded in paraffin, and 8 μm sections were cut for all subsequent stains. Adult skulls were decalcified in 10 % EDTA (w/v) for 96–120 h. Alcian Blue (cartilage) and Alizarin Red (bone) staining [7], light dissection microscope imaging [15] and all other histological stains [16] were conducted as described previously.

### Immunohistochemistry

All Immunohistochemistry experiments were conducted using either 4 % PFA or formalin-fixed, paraffin-embedded tissue, processed and stained using standard methods, as described previously [12, 17]. The antibodies used were *FGFR1* (My Biosource; cat# MBS9209911), *pFGFR1* (Abcam; ab59194), *FGFR2* (My Biosource; cat# MBS821068), *Noggin* (Abcam, ab16054), *Twist* (Santa Cruz, sc-6269), and *Runx2* (Abcam; ab23981), using standard histological methods [12, 17] and according to the manufacturer’s recommendations.

## Results

### Loss of *Grhl3* leads to gross morphological defects and closer apposition of frontal and parietal bones in the E18.5 embryonic skull

**Fig. 1:**
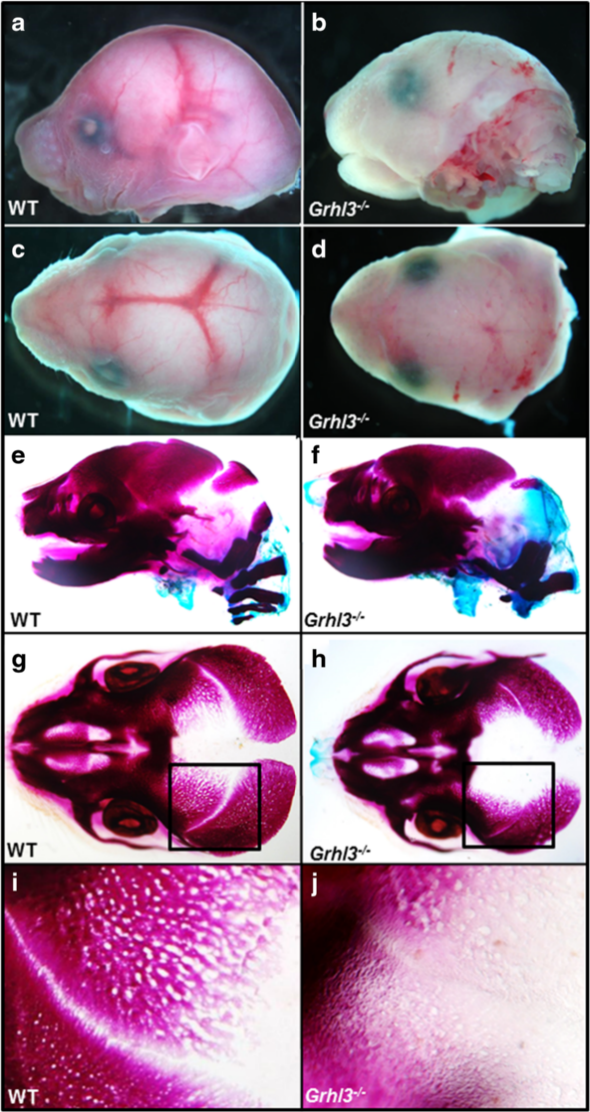
Loss of *Grhl3* leads to premature suture fusion and aberrant skull morphology in E18.5 mouse embryos. **a**-**d** Dissecting light microscopy images showing shortened and flattened skull morphology, lack of suture vascularisation and decreased skull size in *Grhl3* ^*−/−*^ embryos (**b**, **d**) compared to WT (**a**, **c**) at E18.5. **a**, **b** shown in lateral view, C-D in dorsal view. **e**-**h** Alcian Blue/Alizarin red staining of WT (**e**, **g**) and *Grhl3* ^*−/−*^ (**f**, **h**) embryos in lateral (**e**, **f**) and dorsal (**g**, **h**) views, highlighting skull morphology and suture development. **i**, **j** High-powered images of the boxed region in (**g**, **h**), showing a clearly open suture in WT embryos (**i**), and a fused suture in *Grhl3* ^*−/−*^ embryos (**j**)

In order to assess gross developmental phenotypes, E18.5 embryos were collected and viewed by dissecting microscopy. Viewed both laterally (Fig. 1a, b) and dorsally (Fig. 1c, d) the *Grhl3* ^*−/−*^ embryos were noted to have flattened foreheads, elevated, angulated orbital rims and a widened, flatter snout in comparison to control, (wild-type; WT) littermates. Defects in skull morphology were also readily observable following skeletal staining (Fig. 1e, f). Overall, the heads of *Grhl3* ^*−/−*^ embryos appeared shorter, and displayed features highly consistent with human brachycephaly.

In order to more closely examine the ultrastructure of the skull, further analysis of the cranial bones was conducted by skeletal staining. When viewed laterally, a clear demarcation between the frontal and parietal bones at the coronal suture could be seen in WT embryos. The distinction between the two skull bones was less apparent in the *Grhl3* ^*−/−*^ embryo which appeared more flat superiorly. Having dissected the caudal half of the skull from the cranial half to allow clear views of the coronal suture from above we could demonstrate a clear boundary of non-calcified tissue between the frontal and parietal bones in the WT, while this was qualitatively less appreciable in the *Grhl3* ^*−/−*^ (Fig. 1g, h). Magnification of the coronal suture highlighted this boundary further in WT while the *Grhl3* ^*−/−*^ mice presented with a continuum of tissue extending across the suture (Fig. 1i, j). Taken together, these data indicate that loss of *Grhl3* impacts on skull shape, size and apposition of the frontal and parietal bones.

### Micro CT analyses indicate significant differences in cranial dimensions of *Grhl3*^*−/−*^ E18.5 embryos

**Fig. 2:**
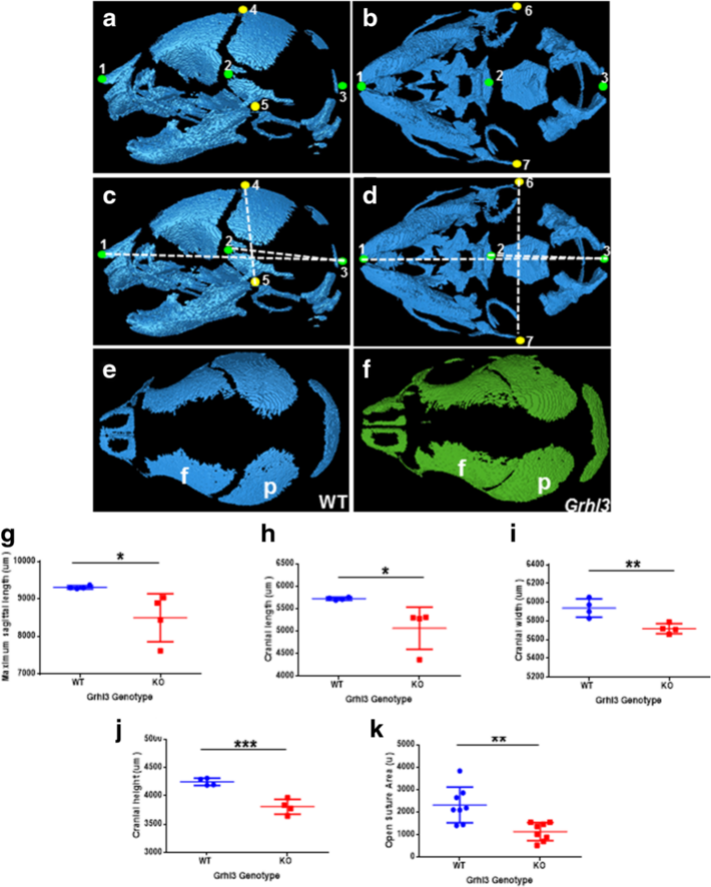
Loss of *Grhl3* leads to significant differences in skull length and width. **a**-**d** Schematic diagrams on WT skulls showing measurements taken using Micro CT analysis. Micrographs highlighting the morphometric landmarks utilised for quantitative analyses, shown in sagittal (**a**) and ventral (**b**) views (see methods for details). Representative diagrams showing measurements taken, namely sagittal length (1–3), cranial vault length (2–3) and width (6–7), and cranial height (4–5; **c**, **d**). **e**, **f** Representative 2D images generated of the skull with the caudal sections removed digitally, allowing clear visualisation of the frontal (**f**) and parietal (p) bones, and the suture between them. (**g**-**k**) Quantitation of measurements of regions outlined in (**a**-**d**), namely maximal sagittal length (**g**), cranial length (**h**), maximal cranial width (**i**), cranial height (**j**), and the open suture area (**k**) in WT and *Grhl3* ^*−/−*^ embryos. *p < 0.05; **p < 0.01; ***p < 0.005

Micro CT allows extremely accurate imaging of the skull bones, even in the relatively undeveloped E18.5 embryo. We performed detailed measurements of both embryonic and adult female mouse skulls, using morphometric landmarks described previously ([14], see methods) and generated images of the skull with the caudal sections removed digitally, allowing clear visualisation of the frontal and parietal bones and measurement of the coronal suture area (Fig. 2a–f; see also 3D rotating movies in Additional file 1: Figure S1). Analyses of the Micro CT measurements showed statistically significantly reduced total sagittal length of the skull, cranial length, cranial width and cranial height in the *Grhl3* ^*−/−*^ embryos compared to the WT controls (Fig. 2g–k). No statistically significant differences were seen in the size of the occipital bone (data not shown). Facial skeleton lengths were also not significantly different between groups, suggesting the overall shortage in skull length was from the shorter cranial length. Measuring coronal suture area on both sides of each embryo (*n* = 8 WT and *n* = 8 *Grhl3* ^*−/−*^) revealed a significant reduction in open suture area in the *Grhl3* ^*−/−*^ embryos compared to their WT littermates, suggesting greater levels of calcification in the *Grhl3* ^*−/−*^ sutures. Compensatory widening of the sagittal suture was seen in 50 % of the embryos, as is often the case in human bicoronal craniosynostosis. These measurements allowed us to quantitatively demonstrate suture openings in the *Grhl3* ^*−/−*^ embryos, and further support the hypothesis of closer apposition of frontal and parietal bones.

### *Grhl3*^*−/−*^ E18.5 embryo skulls present with organised collagen deposition and premature osteoblast alignment, although expression of known genes involved in craniosynostosis is largely unchanged

**Fig. 3:**
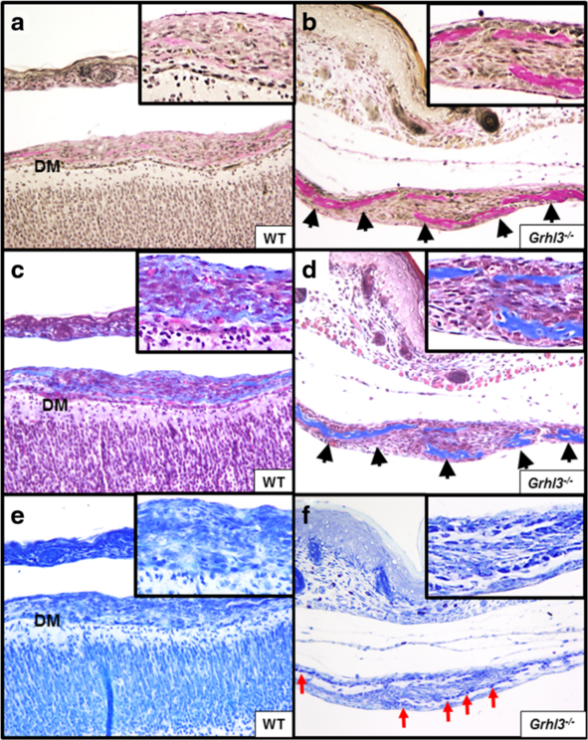
Premature collagen deposition, mineralisation and osteoblast alignment are seen in E18.0 *Grhl3* ^*−/−*^ embryos (**a**, **b**) Verhoeff’s van Gieson’s (collagen) stain showing little evidence of organised collagen deposits at the location of the coronal suture in WT embryos (**a**), in contrast to *Grhl3* ^*−/−*^embryos (arrowheads, **b**). **c**, **d** Masson’s trichrome stain showing diffuse staining in the region of the developing coronal suture in WT embryos (**c**), compared to overlapping projections of blue staining (indicative of increased mineralisation) in *Grhl3* ^*−/−*^ (arrowheads, **d**). **e**, **f** Toluidine blue staining showing dark blue nuclei to define a clear row of osteoblasts along the emerging front in *Grhl3* ^*−/−*^ embryos (red arrows, **f**); these were not noticeable in WT embryos (**e**). Inset (boxed) regions in all panels show increased magnifications of the embryonic skull. Note that the dura mater (DM) and underlying cerebrum are in clear contact with the skull in WT (**a**, **c**, **e**) but not *Grhl3* ^*−/−*^ (**b**, **d**, **f**) embryonic skulls

In order to examine whether the sutures of *Grhl3* ^*−/−*^ embryo skulls displayed histological and molecular hallmarks of a predisposition to premature suture fusion, we firstly examined coronal sections using traditional histological stains (Verhoeff’s van Gieson’s, Masson’s Trichrome, Toluidine blue and Von Kossa stains), which remain both the simplest and clearest method of demonstrating the developing sutures. Sagittal sections of skull from WT and *Grhl3* ^*−/−*^ E18 embryos were stained and compared. Osteogenic fronts of the coronal and lambdoidal sutures normally overlap as they develop, whereas the other sutures about each other before fusing. Type I collagen is the main structural protein in developing bone. It provides the extracellular matrix (ECM) or “scaffold” on which the cellular components of developing cartilage and bone can anchor and gain contextual signals for proliferation and differentiation. Verhoeff’s van Gieson’s stain highlights collagen as red/pink in histological sections. We saw little evidence of organised collagen deposits at the location of the coronal suture in WT E18 embryos, however, in the *Grhl3* ^*−/−*^embryos, clearly overlapping areas of organised collagen can be seen (Fig. 3a, b). Next, we used Masson’s trichrome stain to detect osteoid (developing) bone (which stains red) as well as existing bone (blue). Our data show that the bone profile in WT embryos showed diffuse blue and red staining in the region of the developing coronal suture, whereas the *Grhl3* ^*−/−*^ skulls demonstrated clear overlapping projections of blue staining, suggesting this bone was further mineralised than in the WT controls (Fig. 3c, d). Next, we employed Toluidine blue staining to clearly demarcate the cellular components of newly developing bone, particularly osteoblasts. In our E18 embryo sections, little evidence of organised cell behaviour was visible in the WT skulls, however, in the *Grhl3* ^*−/−*^ animals, the developing osteoid stained dark blue nuclei, to define a clear row of osteoblasts along the emerging front (Fig. 3e, f). Lastly, we used Von Kossa staining to distinguish cartilage from calcified bone, however we could detect no difference in WT and *Grhl3* ^*−/−*^ embryos (data not shown), suggesting that the premature fusion we see is not due to hyper-calcification of the skull. Similar analyses of E18.5 WT, Heterozygous (*Grhl3* ^*+/−*^) and *Grhl3* ^*−/−*^ embryo skulls similarly did not detect any significant differences in mineralisation, collagen deposition or osteoblast alignment between WT and *Grhl3* ^*+/−*^ skulls (Additional file 2: Figure S3).

Previous work had shown that the dura mater, the outer-most membrane enveloping the brain and spinal cord, is critical for the regulation of spatiotemporal cranial suture fusion, putatively through the secretion of paracrine factors [18–20]. We noted in our histological sections that in the majority of WT (and also *Grhl3* ^*+/−*^, not shown) embryos examined, the cerebrum, and therefore dura mater, is clearly visible in close proximity to the bones of the developing skull (Fig. 3a, c, and e). Similarly, the overlying dermis and subcutaneous tissue in WT and *Grhl3* ^*+/−*^ skulls was clearly visible in close contact in our histological sections. However, we noted a significant disconnect between the generally underdeveloped cerebrum and the skull in all *Grhl3* ^*−/−*^ embryos, indicating that the dura mater may not make strong physical contact with the overlying bone (Fig. 3b, d, f); furthermore, we also noted a disconnect in apposition between the overlying dermis and the skull. These results suggest that decreased dorso-ventral tensile strength between both the overlying dermis, and underlying dura, may partially underpin the differential (closer) apposition of frontal and parietal bones seen in *Grhl3* ^*−/−*^ mice.

Lastly, in order to determine whether aberrant regulation of genes known to be involved in the aetiology of craniosynostosis underpinned any of the defects we observed, we examined the expression of *Noggin*, *FGFR1/pFGFR1, FGFR2, Runx2* and *Twist1* by immunohistochemistry (see methods). Save for a potential loss-of-expression of *pFGFR1* in the suprabasal epidermal layers of *Grhl3* ^*−/−*^ mice, we could detect no significant differences in expression of any of these factors in the developing sutures, dermis or dura mater in *Grhl3* ^*−/−*^ embryos, at either E16.5 (Additional file 3: Figure S4a-b) or E18.5, (Additional file 4: Figure S5a-b), indicating that aberrant expression or localisation of these factors is unlikely to account for the phenotypes we observe.

### Heterozygous loss of *Grhl3* leads to defects in skull size, but not premature suture fusion, in adult mice

As noted, the *Grhl3* ^*−/−*^ embryos are lethal at birth; we were therefore unable to analyse cranial morphology and suture fusion in these mice post-natally. However, *Grhl3* ^*+/−*^ mice (both adults and embryos) present with no observable phenotypic defects, and are both viable and fertile. Previous analysis of both *Grhl2* and *Grhl3* function in a variety of animal models, both by us and others, clearly indicates that animals with compromised, but still present, *Grhl3* function (termed “altered gene-dosage”) present with a spectrum of phenotypic defects [8, 21–23]. An example of a hypomorphic *Grhl3* phenotype is the “curly-tail” mutant, in which a small proportion of mice present with a curled-tail and sacral spina bifida due to a mutation in an upstream *Grhl3* regulatory element [24, 25]. Additionally, the homeostatic roles played by this family are often not apparent until later in life, as is the case with *Grhl2* loss ultimately leading to age-related hearing impairment [26]. Lastly, human craniosynostosis itself presents as a spectrum of penetrance and severity of the condition. The subtler deformities may not require intervention, or even be clinically apparent, without careful radiological examination.

**Fig. 4:**
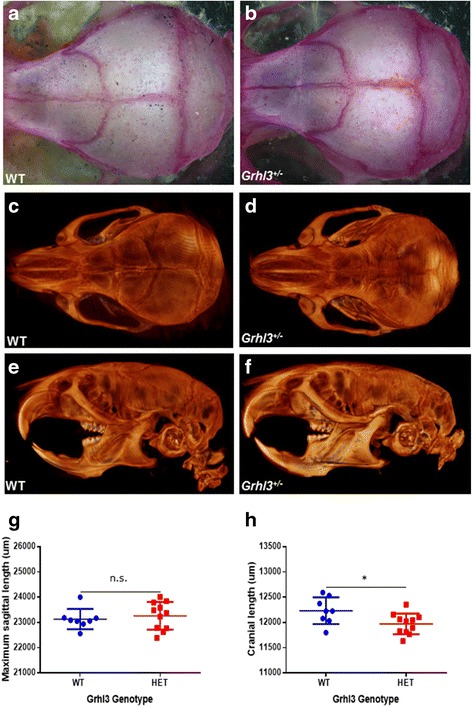
Adult skull analysis in *Grhl3* heterozygous (*Grhl3* ^*+/−*^) mice (**a**, **b**) Light microscopy showing no significant difference in suture size, width between WT (**a**) and *Grhl3* ^*+/−*^ (**b**) skulls. **c**-**f** Micro CT images showing skulls of both WT (**c**, **e**) and *Grhl3* ^*+/−*^ (**d**, **f**) adult mice, in dorsal (**c**, **d**) and lateral (**e**, **f**) views. **g**, **h** Quantification of skull size shown no significant difference (*p* = 0.57) in the maximum sagittal length (**g**), but a statistically significant reduction in cranial length (**h**) between WT and *Grhl3* ^*+/−*^skulls. *p < 0.05

Taking these factors into account, we examined the skulls of adult WT and *Grhl3* ^*+/−*^ skulls, in order to carefully examine suture formation and cranial size. Our gross examinations did not elucidate any obvious abnormalities of the coronal suture or other features (Fig. 4a, b). Using Micro CT, we examined the skulls in order to determine whether subtle defects were present in our heterozygous mouse population, however comparing Micro CT generated images of the skulls both in dorsal (Fig. 4c, d) and lateral (Fig. 4e, f) planes did not demonstrate any significant differences (also see movies in Additional file 5: Figure S2). Quantitative measurements of the maximum sagittal length of the skulls showed no significant difference (Fig. 4g), however, *Grhl3* ^*+/−*^ mice showed a small but statistically significant difference in the length of the cranial cavity (Fig. 4h). Cranial cavity width and height were not statistically different between the two groups (data not shown). Together, these data indicate that *Grhl3* heterozygosity impacts on the size of the cranial cavity, but not suture fusion.

## Discussion

Craniosynostosis is a relatively common congenital deformity affecting the human skull, underpinned by (e.g.) a closer apposition of the frontal and parietal bones. Following the observation that *Grhl3* ^*−/−*^ embryos present with a grossly abnormally skull shape and smaller size than WT littermates, we investigated this phenotype further using Micro CT, and found differences in overall skull length, as a consequence of reduced cranial cavity length. Cranial height and width were also significantly reduced in the *Grhl3* ^*−/−*^ embryos. Histologically, we detected an increased collagen framework deposition in the coronal suture of the *Grhl3* ^*−/−*^ embryos at E18. The *Grhl3* ^*−/−*^ mice coronal sutures also showed a greater degree of mineralisation and organisation of osteoblasts. Interestingly, although not grossly abnormal, the skulls of adult *Grhl3* ^*+/−*^ mice were statistically shorter in terms of length of the cranial compartment, a phenotype which may perhaps mirror some of the sub-clinical human presentations. Our data indicate that *Grhl3* ^*−/−*^ mice are a novel model for intrauterine events leading to the increased apposition of calvarial bones, and our future work will be focused on identifying the underlying genetic mechanisms which underpin this phenotype.

Skull defects in our model are highly consistent with the overall shortened, squat stature of embryos lacking *Grhl3*, due to decreased convergence-extension movements, although interestingly these mice are technically not “smaller”, as the size of other skeletal components, such as the overall length of the spine or limbs, are not significantly smaller in *Grhl3* ^*−/−*^ embryos [7]. These data suggest that head and skull size, as well as suture apposition, may be regulated by cranial-specific *Grhl3* function. Our previous histological examinations characterising the expression of *Grhl3* by in situ hybridisation did not identify expression at the coronal suture site, or within the skeletal precursor cells themselves [27], save for a thin band of expression visible in the anterior-most neurocranium, overlying the olfactory epithelium [7]. However, the expression of *Grhl3* in this region is unlikely to account for the closer apposition of the frontal and parietal bones or decreased skull size we observed. More likely, epithelial extension defects in the overlying surface ectoderm (where *Grhl3* is robustly expressed) lead to secondary defects in the assembly, expansion and temporal regulation of skull development and fusion, whereby the skull is compressed due to decreased availability of space in which to grow.

Closer apposition of future suture sites may lead to premature fusion, as seen in craniosynostosis. Genetic deletion of factors in the epidermis has previously been described to result in defective formation of underlying bone. Deletion of the transcription factor IRF6 (itself an upstream regulator of *Grhl3*; [28]), resulted in significant skin tightening in the limb buds, to the point where the underlying digits are deformed and the limb buds remained as “stumps” [29], whereas deletion of the IkB kinase-a (IKK-α) led to significant craniofacial defects, which interestingly, could be rescued upon restoration of IKK-α re-expression within the epidermis [30]. These data suggest that genetic defects during development may manifest through a putative disruption of epidermal-mesodermal, or epidermal-neural crest interactions, resulting in subsequent patterning and morphogenesis of the bone.

A second possibility pertains to a disruption to the identity and fate of the neural crest. Although the coronal suture progenitor cells are generally thought to be *Sonic hedgehog* (*Shh*)-responsive cells of paraxial mesodermal origin [31, 32], the frontal bone itself is largely neural-crest derived, suggesting that mislocalisation of the neural crest within the boundary may contribute to defective establishment of a clear demarcation (boundary) at the neural-crest/mesoderm interface of the future frontal/parietal suture. This precise phenotype has in fact been described previously in the context of disrupted *Ephrin-Eph* signalling downstream of aberrant *Msx2* or *Twist1* signalling [5]. Coupled with a recent study showing that *Grhl3* is expressed at the neural plate/non-neural ectoderm border, the site of future neural crest cell delamination and migration [33], one could imagine that a failure in instructional cues governing neural crest cell fate, particularly a shift in the signals required for maintenance of positioning at the suture interface, may contribute to some of the subsequent patterning and morphological defects we observe, Our current studies are aimed at addressing which of these mechanisms is likely to underpin the closer apposition of the frontal and parietal bones we observe in *Grhl3* ^*−/−*^ embryos, particularly by investigating the cell-intrinsic role played by *Grhl3* within the neural crest, closer investigation of *Ephrin-Eph* signalling in the early stages of embryogenesis, putative links between *Shh* and *grhl3* [34], and modelling *grhl3* deletion in the surface ectoderm (and subsequent skull development) using the highly tractable zebrafish model system.

Further supporting our model that the closer apposition of the frontal and parietal bones we observe is secondary, rather than primary (i.e. cell autonomous), is previous evidence regarding the important role played by the dura mater on maintaining cranial sutures. Previous transplant experiments [35, 36] examined the requirement of the dura mater in the development and maintenance of the normally patent coronal suture by transplanting coronal suture complexes with or without dura mater from E19 and P1 rats to attempt rescue of parietal defects in adult rats. In the absence of dura mater, the coronal suture was fused prematurely by 3 weeks after transplantation, whereas in the presence of dura mater, the coronal suture remained open. The mechanisms for regulation were thought to be a secretion of necessary paracrine factors, particularly members of the FGF, IGF and TGFβ families of mitogens [37–40] from the dura mater to the osteoblasts of the developing sutures. Although we have not detected expression of *Grhl3* in the dura mater [27], and therefore a dura-specific defect is unlikely, we speculate that the reduced adhesive strength between the dura and cranial suture sites, resulting from defective convergence-extension as discussed above, may also be a contributing factor to the closer apposition of the frontal and parietal bones we observe.

As *Grhl3* functions as a transcription factor, whose role is to activate or repress the expression of target genes through direct binding to promoter or enhancer sequences, we also investigated putative regulatory mechanisms which may contribute to our observed phenotype. We had previously generated a list of 305 candidate target genes, based on alignment of gene promoters across placental mammals, and interrogating these for the presence of the conserved *Grhl*-family binding site, broadly AA**C**CG**G**TT (with the first “C” and second “G” invariant). Our group has previously identified numerous genes from this list as true *Grhl*-target genes, including *Tgm1* [9]*, Dsg1* [41]*, PTEN* [12]*, RhoGEF19* [10]*, eng2a* and *spec1* [21]*, edn1* [22] and *GSK3β* [17]. From this list, we identified another potential candidate, *FGFR1*. Mutations in the *FGFR* family are known to cause craniosynostosis in various human syndromes [42], largely through an aberrant balance between apoptosis and proliferation of suture cells, making *FGFR1* a logical potential target for investigation of the mechanism causing craniosynostosis in our model. However, neither our immunohistochemistry experiments, or qPCR of suture tissue dissected from *Grhl3* embryos (data not shown) detected a significant difference in expression between WT and *Grhl3* ^*−/−*^ embryos in either the dura, calvaria or the suture site. Interestingly, we did note an apparent decrease of activated *pFGFR1* in the most superficial layers of the epidermis, overlying the skull, potentially suggestive of changes in epidermal fidelity (Additional file 3: Figure S4, Additional file 4: Figure S5). As *Grhl3* and *FGFR1* are co-expressed within the overlying surface ectoderm, our future studies will further investigate this relationship in the regulation of epidermal development and function.

Lastly, we also investigated the expression of multiple other factors, previously described as being involved in suture closure and skull development (*Noggin, Twist, Runx2* and *FGFR2*), but could not discern any differences in expression of any of these, within the epidermis, suture sites, calvaria outside the sutures or in the dermis. Taken together with the lack of *Grhl3* expression within the suture site, brain or dermis, these data further supporting our theory that closer apposition of the frontal and parietal bones in our model is not caused by cell-intrinsic defects within the cranial osteoblasts, but rather is a secondary consequence of either ectodermal tension or correct neural crest patterning, homing and fidelity.

## Conclusions

Coronal craniosynostosis in humans presents with a spectrum of severity and its aetiology is multifactorial outside a narrow set of syndromes. We have shown by Micro CT and histological techniques that genetic loss of *Grhl3* in our mouse model results in closer apposition of the frontal and parietal bones, mimicking the early stages of craniosynostosis which may cause significant problems for the animal were it able to survive postnatally. The role of *Grhl3*, identification of binding partners and interaction with downstream target genes during development forms a promising avenue of investigation to help better understand the mechanisms by which closer apposition of the frontal and parietal bones, and possibly also coronal craniosynostosis, develops.

## Additional files

Additional file 1: Figure S1. 3D rotating movies of representative E18.5 skulls. Representative movies allowing better visualisation of the skull bones and coronal sutures in both WT (A) and *Grhl3* ^*−/−*^ (B) E18.5 embryos. (ZIP 13 mb)
Additional file 2: Figure S3. Analysis of collagen deposition, mineralisation and osteoblast alignment in E18.5 WT, *Grhl3* ^*+/−*^ and *Grhl3* ^*−/−*^ skulls (A-C) Although collagen deposition in WT embryos appears more advanced in E18.5 embryos c.f. E18.0 embryos (Verhoeff’s van Gieson’s stain), organised collagen deposits are clearly visible in *Grhl3* ^*+/−*^ embryos (arrows), and both are less pronounced than in *Grhl3* ^*−/−*^ embryos. (D-F) Masson’s trichrome stain showing little difference in mineralisation between WT and *Grhl3* ^*+/−*^ embryos. (G–I) Toluidine blue staining showing dark blue nuclei (arrows in I) to define a clear row of osteoblasts along the emerging front in *Grhl3* ^*−/−*^ embryos; there is little evidence of this in either WT or *Grhl3* ^*+/−*^ embryos. (TIF 671 kb)
Additional file 3: Figure S4. Analysis of gene expression in the developing coronal sutures of WT and Grhl3−/− embryos at E16.5 (A-B) The expression of β-galactosidase (corresponding to the LacZ transgene inserted within the *Grhl3* locus, simultaneously disrupting gene function, as well as acting as a reporter to confirm presence of the deleted allele [7]) was used to confirm loss of *Grhl3*. The expression of total *FGFR1* (C-D), *pFGFR1* (E-F), *FGFR2* (G-H), *Twist* (I-J), *Runx2* (K-L) and *Noggin* (M-N) was examined by immunohistochemical analysis. no significant differences in the expression of any of these genes was detected, at E16.5 within the calvaria, dura or suture regions. (ZIP 353 kb)
Additional file 4: Figure S5. Analysis of gene expression in the developing coronal sutures of WT and Grhl3−/− embryos at E18.5. (A-B) The expression of β-galactosidase (corresponding to the LacZ transgene inserted within the *Grhl3* locus, simultaneously disrupting gene function, as well as acting as a reporter to confirm presence of the deleted allele [7]) was used to confirm loss of *Grhl3*. The expression of total *FGFR1* (C-D), *pFGFR1* (E-F), *FGFR2* (G-H), *Twist* (I-J), *Runx2* (K-L) and *Noggin* (M-N) was examined by immunohistochemical analysis. Other than an apparent loss of *pFGFR1* in the most superficial layers of the surface ectoderm in *Grhl3* ^*−/−*^ embryos at E18.5 (compare E with F; arrowheads), no significant differences in the expression of any of these genes was detected at E18.5, within the calvaria, dura or suture regions. (ZIP 362 kb)
Additional file 5: Figure S2. 3D rotating movies of representative adult skulls. Representative movies allowing better visualisation of the skull bones and coronal sutures in both WT (A) and *Grhl3* ^*+/−*^ (B) adult skulls. (ZIP 2 mb)

## References

[1] Di Rocco F, Arnaud E, Renier D (2009). Evolution in the frequency of nonsyndromic craniosynostosis. J Neurosurg Pediatr.

[2] Lajeunie E, Le Merrer M, Bonaiti-Pellie C, Marchac D, Renier D (1995). Genetic study of nonsyndromic coronal craniosynostosis. Am J Med Genet.

[3] Boulet SL, Rasmussen SA, Honein MA (2008). A population-based study of craniosynostosis in metropolitan Atlanta, 1989–2003. Am J Med Genet.

[4] Governale LS (2015). Craniosynostosis. Pediatr Neurol.

[5] Merrill AE, Bochukova EG, Brugger SM, Ishii M, Pilz DT, Wall SA, Lyons KM, Wilkie AO, Maxson RE (2006). Cell mixing at a neural crest-mesoderm boundary and deficient ephrin-Eph signaling in the pathogenesis of craniosynostosis. Hum Mol Genet.

[6] Wilanowski T, Tuckfield A, Cerruti L, O’Connell S, Saint R, Parekh V, Tao J, Cunningham JM, Jane SM (2002). A highly conserved novel family of mammalian developmental transcription factors related to Drosophila grainyhead. Mech Dev.

[7] Ting SB, Wilanowski T, Auden A, Hall M, Voss AK, Thomas T, Parekh V, Cunningham JM, Jane SM (2003). Inositol- and folate-resistant neural tube defects in mice lacking the epithelial-specific factor Grhl-3. Nat Med.

[8] Rifat Y, Parekh V, Wilanowski T, Hislop NR, Auden A, Ting SB, Cunningham JM, Jane SM (2010). Regional neural tube closure defined by the Grainy head-like transcription factors. Dev Biol.

[9] Ting SB, Caddy J, Hislop N, Wilanowski T, Auden A, Zhao LL, Ellis S, Kaur P, Uchida Y, Holleran WM (2005). A homolog of Drosophila grainy head is essential for epidermal integrity in mice. Science (New York).

[10] Caddy J, Wilanowski T, Darido C, Dworkin S, Ting SB, Zhao Q, Rank G, Auden A, Srivastava S, Papenfuss TA (2010). Epidermal wound repair is regulated by the planar cell polarity signaling pathway. Dev Cell.

[11] Hislop NR, Caddy J, Ting SB, Auden A, Vasudevan S, King SL, Lindeman GJ, Visvader JE, Cunningham JM, Jane SM (2008). Grhl3 and Lmo4 play coordinate roles in epidermal migration. Dev Biol.

[12] Darido C, Georgy SR, Wilanowski T, Dworkin S, Auden A, Zhao Q, Rank G, Srivastava S, Finlay MJ, Papenfuss AT (2011). Targeting of the tumor suppressor GRHL3 by a miR-21-dependent proto-oncogenic network results in PTEN loss and tumorigenesis. Cancer Cell.

[13] Schneider CA, Rasband WS, Eliceiri KW (2012). NIH Image to ImageJ: 25 years of image analysis. Nat Methods.

[14] Motch Perrine SM, Cole TM, Martinez-Abadias N, Aldridge K, Jabs EW, Richtsmeier JT (2014). Craniofacial divergence by distinct prenatal growth patterns in Fgfr2 mutant mice. BMC Dev Biol.

[15] Dworkin S, Heath JK, DeJong-Curtain TA, Hogan BM, Lieschke GJ, Malaterre J, Ramsay RG, Mantamadiotis T (2007). CREB activity modulates neural cell proliferation, midbrain-hindbrain organization and patterning in zebrafish. Dev Biol.

[16] Regelsberger J, Milovanovic P, Schmidt T, Hahn M, Zimmermann EA, Tsokos M, Zustin J, Ritchie RO, Amling M, Busse B (2012). Changes to the cell, tissue and architecture levels in cranial suture synostosis reveal a problem of timing in bone development. European cells materials.

[17] Georgy SR, Cangkrama M, Srivastava S, Partridge D, Auden A, Dworkin S, McLean CA, Jane SM, Darido C (2015). Identification of a Novel Proto-oncogenic Network in Head and Neck Squamous Cell Carcinoma. J Natl Cancer Inst.

[18] Bradley JP, Levine JP, Blewett C, Krummel T, McCarthy JG, Longaker MT (1996). Studies in cranial suture biology: in vitro cranial suture fusion. Cleft palate-craniofacial j.

[19] Bradley JP, Levine JP, McCarthy JG, Longaker MT (1997). Studies in cranial suture biology: regional dura mater determines in vitro cranial suture fusion. Plast Reconstr Surg.

[20] Levine JP, Bradley JP, Roth DA, McCarthy JG, Longaker MT (1998). Studies in cranial suture biology: regional dura mater determines overlying suture biology. Plast Reconstr Surg.

[21] Dworkin S, Darido C, Georgy SR, Wilanowski T, Srivastava S, Ellett F, Pase L, Han Y, Meng A, Heath JK (2012). Midbrain-hindbrain boundary patterning and morphogenesis are regulated by diverse grainy head-like 2-dependent pathways. Development (Cambridge, England).

[22] Dworkin S, Simkin J, Darido C, Partridge DD, Georgy SR, Caddy J, Wilanowski T, Lieschke GJ, Doggett K, Heath JK (2014). Grainyhead-like 3 regulation of endothelin-1 in the pharyngeal endoderm is critical for growth and development of the craniofacial skeleton. Mech Dev.

[23] de la Garza G, Schleiffarth JR, Dunnwald M, Mankad A, Weirather JL, Bonde G, Butcher S, Mansour TA, Kousa YA, Fukazawa CF (2012). Interferon Regulatory Factor 6 Promotes Differentiation of the Periderm by Activating Expression of Grainyhead-Like 3. J Invest Dermatol.

[24] van Straaten HW, Copp AJ (2001). Curly tail: a 50-year history of the mouse spina bifida model. Anat Embryol.

[25] Gustavsson P, Copp AJ, Greene ND (2008). Grainyhead genes and mammalian neural tube closure. Birth defects res.

[26] Van Laer L, Van Eyken E, Fransen E, Huyghe JR, Topsakal V, Hendrickx JJ, Hannula S, Maki-Torkko E, Jensen M, Demeester K (2008). The grainyhead like 2 gene (GRHL2), alias TFCP2L3, is associated with age-related hearing impairment. Hum Mol Genet.

[27] Auden A, Caddy J, Wilanowski T, Ting SB, Cunningham JM, Jane SM (2006). Spatial and temporal expression of the Grainyhead-like transcription factor family during murine development. Gene Expr Patterns.

[28] Botti E, Spallone G, Moretti F, Marinari B, Pinetti V, Galanti S, De Meo PD, De Nicola F, Ganci F, Castrignano T (2011). Developmental factor IRF6 exhibits tumor suppressor activity in squamous cell carcinomas. Proc Natl Acad Sci U S A.

[29] Ingraham CR, Kinoshita A, Kondo S, Yang B, Sajan S, Trout KJ, Malik MI, Dunnwald M, Goudy SL, Lovett M (2006). Abnormal skin, limb and craniofacial morphogenesis in mice deficient for interferon regulatory factor 6 (Irf6). Nat Genet.

[30] Sil AK, Maeda S, Sano Y, Roop DR, Karin M (2004). IkappaB kinase-alpha acts in the epidermis to control skeletal and craniofacial morphogenesis. Nature.

[31] Deckelbaum RA, Holmes G, Zhao Z, Tong C, Basilico C, Loomis CA (2012). Regulation of cranial morphogenesis and cell fate at the neural crest-mesoderm boundary by engrailed 1. Development (Cambridge, England).

[32] Ishii M, Sun J, Ting MC, Maxson RE (2015). The Development of the Calvarial Bones and Sutures and the Pathophysiology of Craniosynostosis. Curr Top Dev Biol.

[33] Kimura-Yoshida C, Mochida K, Ellwanger K, Niehrs C, Matsuo I (2015). Fate Specification of Neural Plate Border by Canonical Wnt Signaling and Grhl3 is Crucial for Neural Tube Closure. EBioMedicine.

[34] Dworkin SB, Y, Owens, H, Goldie, SJ. The Role of Sonic Hedgehog in Craniofacial Patterning, Morphogenesis and Cranial Neural Crest Survival. J Dev Biol. 2016;4(3). http://www.mdpi.com/2221-3759/4/3/24/html. 10.3390/jdb4030024 PMC5831778 29615588

[35] Opperman LA (2000). Cranial sutures as intramembranous bone growth sites. Dev Dyn.

[36] Mooney MP, Burrows AM, Smith TD, Losken HW, Opperman LA, Dechant J, Kreithen AM, Kapucu R, Cooper GM, Ogle RC (2001). Correction of coronal suture synostosis using suture and dura mater allografts in rabbits with familial craniosynostosis. Cleft palate-craniofacial j.

[37] Bradley JP, Han VK, Roth DA, Levine JP, McCarthy JG, Longaker MT (1999). Increased IGF-I and IGF-II mRNA and IGF-I peptide in fusing rat cranial sutures suggest evidence for a paracrine role of insulin-like growth factors in suture fusion. Plast Reconstr Surg.

[38] Gosain AK, Recinos RF, Agresti M, Khanna AK (2004). TGF-beta1, FGF-2, and receptor mRNA expression in suture mesenchyme and dura versus underlying brain in fusing and nonfusing mouse cranial sutures. Plast Reconstr Surg.

[39] Kim HJ, Rice DP, Kettunen PJ, Thesleff I (1998). FGF-, BMP- and Shh-mediated signalling pathways in the regulation of cranial suture morphogenesis and calvarial bone development. Development (Cambridge, England).

[40] Spector JA, Greenwald JA, Warren SM, Bouletreau PJ, Detch RC, Fagenholz PJ, Crisera FE, Longaker MT (2002). Dura mater biology: autocrine and paracrine effects of fibroblast growth factor 2. Plast Reconstr Surg.

[41] Wilanowski T, Caddy J, Ting SB, Hislop NR, Cerruti L, Auden A, Zhao LL, Asquith S, Ellis S, Sinclair R (2008). Perturbed desmosomal cadherin expression in grainy head-like 1-null mice. Embo J.

[42] Robin NH, Falk MJ, Haldeman-Englert CR. FGFR-Related Craniosynostosis Syndromes. In: GeneReviews(R). Edited by Pagon RA, Adam MP, Ardinger HH, Wallace SE, Amemiya A, Bean LJH, Bird TD, Fong CT, Mefford HC, Smith RJH et al. Seattle: University of Washington; 1993.

