# Supplementary figures and images for: Mice lacking the conserved transcription factor Grainyhead-like 3 (Grhl3) display increased apposition of the frontal and parietal bones during embryonic development
Source: BMC Dev Biol. 2016 Oct 18;16:37. doi: 10.1186/s12861-016-0136-7 (PMC5070091; doi:10.1186/s12861-016-0136-7)

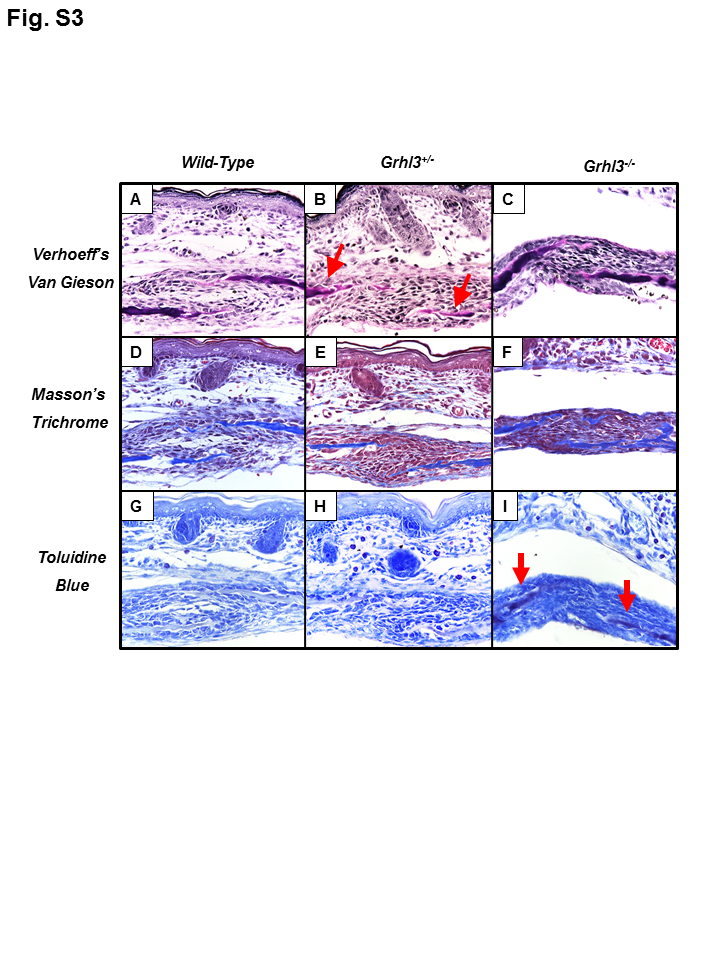

Supplement: Additional file 2: Figure S3. — Analysis of collagen deposition, mineralisation and osteoblast alignment in E18.5 WT, Grhl3 +/− and Grhl3 −/− skulls (A-C) Although collagen deposition in WT embryos appears more advanced in E18.5 embryos c.f. E18.0 embryos (Verhoeff’s van Gieson’s stain), organised collagen deposits are clearly visible in Grhl3 +/− embryos (arrows), and both are less pronounced than in Grhl3 −/− embryos. (D-F) Masson’s trichrome stain showing little difference in mineralisation between WT and Grhl3 +/− embryos. (G–I) Toluidine blue staining showing dark blue nuclei (arrows in I) to define a clear row of osteoblasts along the emerging front in Grhl3 −/− embryos; there is little evidence of this in either WT or Grhl3 +/− embryos. (TIF 671 kb) [file 12861_2016_136_MOESM2_ESM.tif]

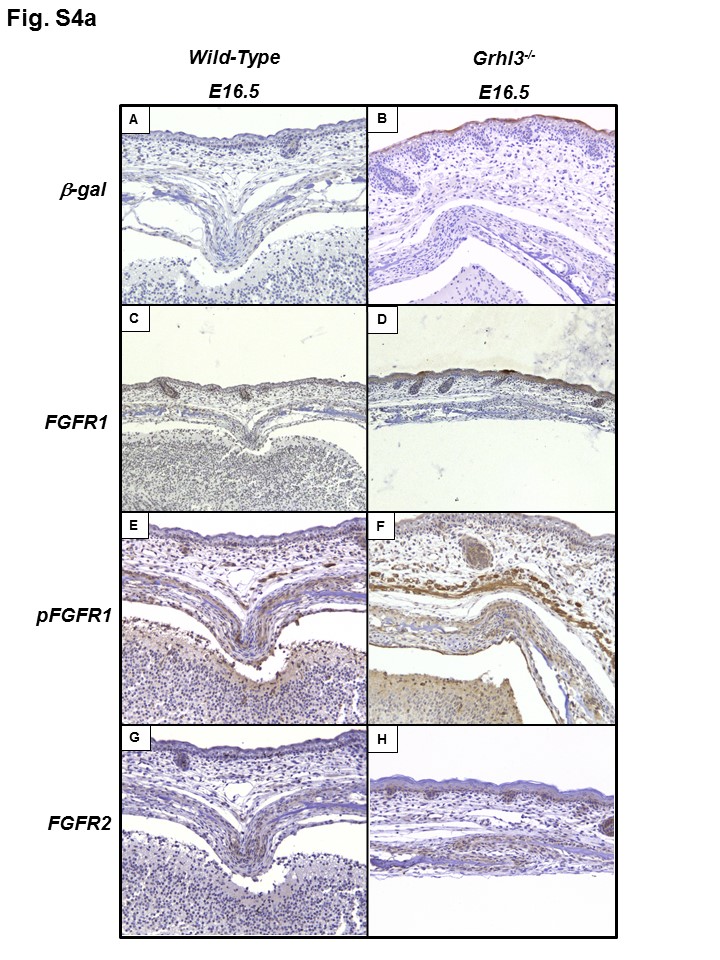

Supplement: Additional file 3: Figure S4. — Analysis of gene expression in the developing coronal sutures of WT and Grhl3−/− embryos at E16.5 (A-B) The expression of β-galactosidase (corresponding to the LacZ transgene inserted within the Grhl3 locus, simultaneously disrupting gene function, as well as acting as a reporter to confirm presence of the deleted allele [7]) was used to confirm loss of Grhl3. The expression of total FGFR1 (C-D), pFGFR1 (E-F), FGFR2 (G-H), Twist (I-J), Runx2 (K-L) and Noggin (M-N) was examined by immunohistochemical analysis. no significant differences in the expression of any of these genes was detected, at E16.5 within the calvaria, dura or suture regions. (ZIP 353 kb) [file 12861_2016_136_MOESM3_ESM.zip › Dworkin_Fig.S4A.JPG]

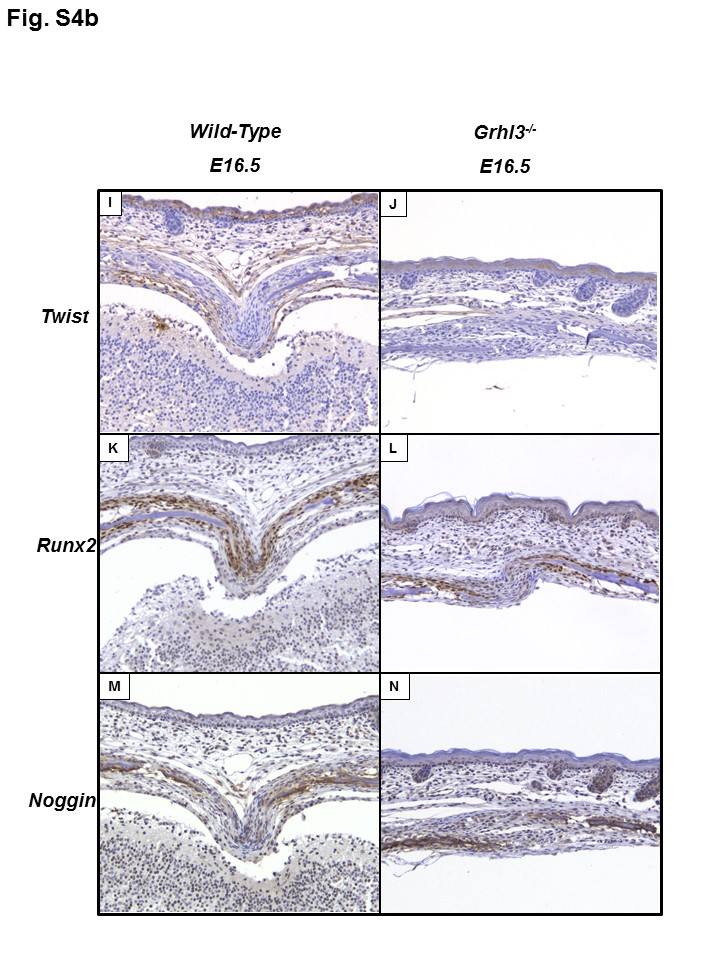

Supplement: Additional file 3: Figure S4. — Analysis of gene expression in the developing coronal sutures of WT and Grhl3−/− embryos at E16.5 (A-B) The expression of β-galactosidase (corresponding to the LacZ transgene inserted within the Grhl3 locus, simultaneously disrupting gene function, as well as acting as a reporter to confirm presence of the deleted allele [7]) was used to confirm loss of Grhl3. The expression of total FGFR1 (C-D), pFGFR1 (E-F), FGFR2 (G-H), Twist (I-J), Runx2 (K-L) and Noggin (M-N) was examined by immunohistochemical analysis. no significant differences in the expression of any of these genes was detected, at E16.5 within the calvaria, dura or suture regions. (ZIP 353 kb) [file 12861_2016_136_MOESM3_ESM.zip › Dworkin_Fig.S4B.JPG]

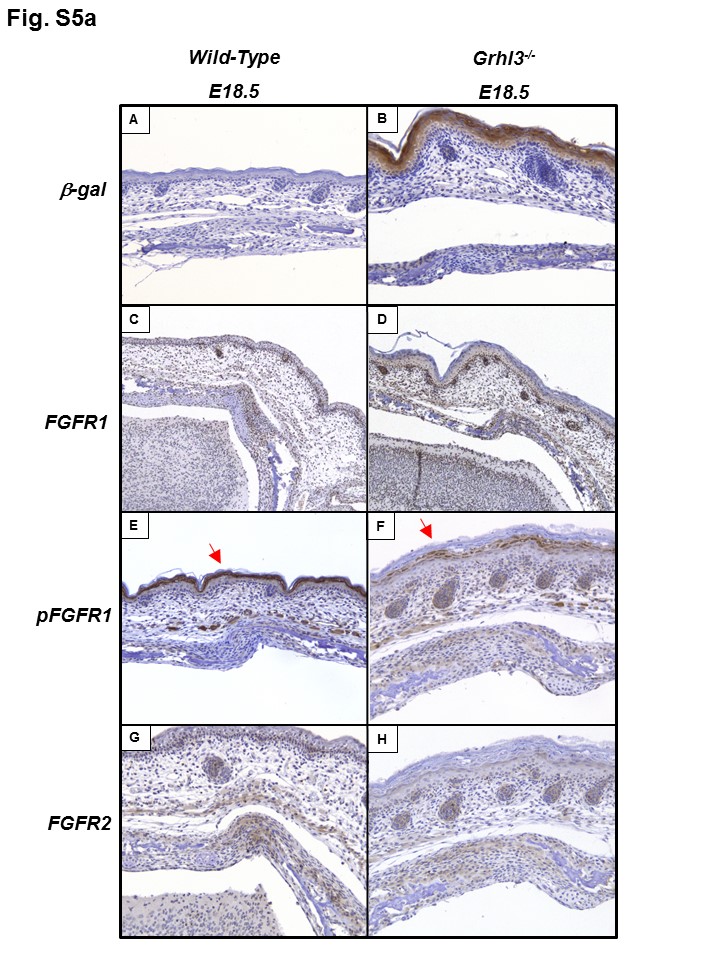

Supplement: Additional file 4: Figure S5. — Analysis of gene expression in the developing coronal sutures of WT and Grhl3−/− embryos at E18.5. (A-B) The expression of β-galactosidase (corresponding to the LacZ transgene inserted within the Grhl3 locus, simultaneously disrupting gene function, as well as acting as a reporter to confirm presence of the deleted allele [7]) was used to confirm loss of Grhl3. The expression of total FGFR1 (C-D), pFGFR1 (E-F), FGFR2 (G-H), Twist (I-J), Runx2 (K-L) and Noggin (M-N) was examined by immunohistochemical analysis. Other than an apparent loss of pFGFR1 in the most superficial layers of the surface ectoderm in Grhl3 −/− embryos at E18.5 (compare E with F; arrowheads), no significant differences in the expression of any of these genes was detected at E18.5, within the calvaria, dura or suture regions. (ZIP 362 kb) [file 12861_2016_136_MOESM4_ESM.zip › Dworkin_Fig.S5A.JPG]

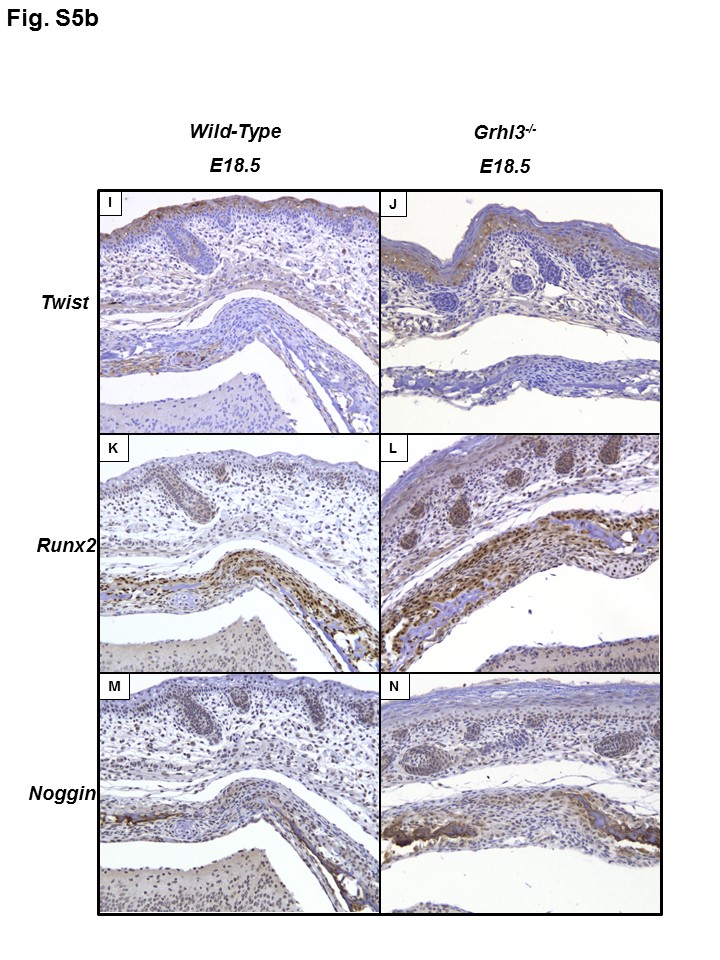

Supplement: Additional file 4: Figure S5. — Analysis of gene expression in the developing coronal sutures of WT and Grhl3−/− embryos at E18.5. (A-B) The expression of β-galactosidase (corresponding to the LacZ transgene inserted within the Grhl3 locus, simultaneously disrupting gene function, as well as acting as a reporter to confirm presence of the deleted allele [7]) was used to confirm loss of Grhl3. The expression of total FGFR1 (C-D), pFGFR1 (E-F), FGFR2 (G-H), Twist (I-J), Runx2 (K-L) and Noggin (M-N) was examined by immunohistochemical analysis. Other than an apparent loss of pFGFR1 in the most superficial layers of the surface ectoderm in Grhl3 −/− embryos at E18.5 (compare E with F; arrowheads), no significant differences in the expression of any of these genes was detected at E18.5, within the calvaria, dura or suture regions. (ZIP 362 kb) [file 12861_2016_136_MOESM4_ESM.zip › Dworkin_Fig.S5B.JPG]
